# The sea urchin *Lytechinus variegatus* lives close to the upper thermal limit for early development in a tropical lagoon

**DOI:** 10.1002/ece3.2317

**Published:** 2016-07-17

**Authors:** Rachel Collin, Kit Yu Karen Chan

**Affiliations:** ^1^ Smithsonian Tropical Research InstituteApartado Postal 0843‐03092Balboa AnconPanama; ^2^ Division of Life ScienceHong Kong University of Science and TechnologyClear Water BayHong KongHong Kong

**Keywords:** Caribbean, global warming, pluteus, safety factor, seagrass, upper thermal limit

## Abstract

Thermal tolerance shapes organisms' physiological performance and limits their biogeographic ranges. Tropical terrestrial organisms are thought to live very near their upper thermal tolerance limits, and such small thermal safety factors put them at risk from global warming. However, little is known about the thermal tolerances of tropical marine invertebrates, how they vary across different life stages, and how these limits relate to environmental conditions. We tested the tolerance to acute heat stress of five life stages of the tropical sea urchin *Lytechinus variegatus* collected in the Bahía Almirante, Bocas del Toro, Panama. We also investigated the impact of chronic heat stress on larval development. Fertilization, cleavage, morula development, and 4‐armed larvae tolerated 2‐h exposures to elevated temperatures between 28–32°C. Average critical temperatures (LT_50_) were lower for initiation of cleavage (33.5°C) and development to morula (32.5°C) than they were for fertilization (34.4°C) or for 4‐armed larvae (34.1°C). LT_50_ was even higher (34.8°C) for adults exposed to similar acute thermal stress, suggesting that thermal limits measured for adults may not be directly applied to the whole life history. During chronic exposure, larvae had significantly lower survival and reduced growth when reared at temperatures above 30.5°C and did not survive chronic exposures at or above 32.3°C. Environmental monitoring at and near our collection site shows that *L. variegatus* may already experience temperatures at which larval growth and survival are reduced during the warmest months of the year. A published local climate model further suggests that such damaging warm temperatures will be reached throughout the Bahía Almirante by 2084. Our results highlight that tropical marine invertebrates likely have small thermal safety factors during some stages in their life cycles, and that shallow‐water populations are at particular risk of near future warming.

## Introduction

Increased environmental temperature is one of the greatest current threats to global biodiversity. It is therefore a major objective of integrative and comparative physiology to predict patterns of organismal vulnerability to thermal stress. Such prediction requires a comprehensive understanding of vulnerability to thermal stress that can be generalized across regions, habitats, taxa, and life history stages (Munday et al. 2013; Bozinovic and Pörtner 2015; Polgar et al. 2015).

Among terrestrial species, thermal tolerance is often related to environmental conditions. For example, lowland tropical species, which experience relatively stable temperature regimes, have narrow ranges of thermal tolerance between the lower and upper thermal limit (UTL). This suggests that their thermal safety margins (i.e., the difference between the UTL and environmental temperature) are smaller and therefore tropical species are under a greater risk from climate change than their temperate counterparts (Huey 1978; Deutsch et al. 2008; Clusella‐Trullas et al. 2011; Sunday et al. 2011; Huey et al. 2012). Similar patterns have been found in marine organisms (Stillman and Somero 2000; Somero 2005; Sunday et al. 2011). For example, adult bivalves from tropical locations have smaller thermal tolerance ranges and safety margins than do those from temperate sites (Compton et al. 2007). Likewise, shallow‐water invertebrates living in the thermally stable subtidal have lower UTLs than do those living in the more variable intertidal (Stillman and Somero 2000; Nguyen et al. 2011). Although widely comparative across latitude, these studies are limited by the fact that they do not evaluate intraspecific variation in thermal tolerance among populations, individuals, or life stages.

Intraspecific variation in thermal response is important to take into account to make predictions about a species' potential for coping with environmental change (Huey et al. 2012; Moritz et al. 2012; Clusella‐Trullas and Chown 2014; Tangwancharoen and Burton 2014; Simon et al. 2015). Here, we will focus on one potentially important source of intraspecific variation in upper thermal limits, variation among life history stages. The details of thermal tolerance and thermal physiology are generally measured on one life stage, most often focusing on the stage that is most amenable to experimental investigation of performance and stress responses. If, as is generally believed, early developmental stages are more susceptible to stressors than are later stages (Byrne 2012 but see Tangwancharoen and Burton 2014 and Hamdoun and Epel 2007), data from adult stages may not give the full picture of thermal tolerances. In marine invertebrates, free‐living developmental stages may include free spawned eggs and sperm, embryos, and free‐living planktonic larvae. The few published studies demonstrate that different life stages often have different thermal tolerances, but do not provide support for a consistent pattern. Studies of crustaceans have found that larval stages have lower thermal tolerance than adults (e.g., Miller et al. 2013; Schiffer et al. 2014). In at least one species of crab, the late larval stages show less tolerance to thermal stress than do early larval stages (Storch et al. 2011). However, the shallow‐water gastropod *Crepidula fornicata* and the intertidal copepod *Tigriopus californicus* show the opposite pattern, with embryos and early life stages showing a higher tolerance to thermal stress than adults (Diederich and Pechenik 2013; Tangwancharoen and Burton 2014).

We use the common tropical sea urchin *Lytechinus variegatus* to study how thermal tolerance varies across the life cycle and to determine if this species experiences temperatures close to its thermal tolerance. *L. variegatus* is an important herbivore in Caribbean seagrass habitats (Valentine and Heck 1991; Klumpp et al. 1993; Heck and Valentine 1995; Rose et al. 1999) and is of importance in aquaculture (Lawrence 2001; Watts et al. 2001; Buitrago et al. 2005). These urchins have been the subject numerous studies of embryology and the larvae are easy to obtain and have been the subject of a wide range of studies (e.g., Boidron‐Metairon 1988; Roller and Stickle 1993; Summers et al. 1996; Cameron et al. 1985; McEdward, L. R., and J. C. Herrera. 1999; Beddingfield and McClintock 2000; Burdett‐Coutts and Metaxas 2004; George et al. 2004). To understand variation in UTLs over the life cycle, we assayed the lethal response to 2‐h acute thermal stress in early embryos, 4‐armed larvae, and adults. We also assayed the temperatures at which fertilization succeeds, and the impact of chronic stress on larval growth and survival.

## Materials and Methods

Adult *Lytechinus variegatus* were collected from seagrass meadows in the shallow waters around Isla Colon and Isla Solarte, in Bocas del Toro, Panama (Collin 2005; Collin et al. 2005, 2009; D'Croz et al. 2005; Kaufmann and Thompson 2005), with permission from Autoridad de los Recursos Acuáticos de Panamá. Laboratory experiments were conducted at the Smithsonian Tropical Research Institute's Bocas del Toro Research Station.

### Adult tolerance of acute stress

We tested adult tolerance of acute stress by subjecting them to a 2‐h exposure to increased temperature and assaying their responses by timing the righting response (Brothers and McClintock 2015; Sherman 2015) and recording the temperature at which they enter heat coma and ultimately die. Adults were tested within 4 days of collection from the field and prior to the test they were maintained at ambient temperature (28–29°C) in the running seawater system at the Bocas del Toro Research Station. Three adults were assigned to each tank and their righting response time at 28°C was recorded prior to applying the treatment. The temperature was then raised slowly to attain the desired temperature after 15 min. After 2 h, the righting response was timed again and if righting failed the urchin was scored as being in a heat coma. Urchins were given a day to recover at 28–29°C in flow through tanks and then scored as alive or dead. Each temperature was tested three times with three urchins in each trial and no urchins were used in more than one trial. Initially urchins were tested at 28 (control), 30, 32, 34, and 36°C. Because we observed 100% mortality at 36°C and 100% survival at 34°C, we added a treatment at 35°C to obtain a more precise estimate of the lethal temperature. Temperature loggers (ibutton Thermochron DS1922T, Maxim Integrated Co.) were used to record the temperature in each treatment every 5 min during the trials. Cross‐calibration of the ibuttons with a YSI temperature probe and the Omega High Accuracy Digital Thermometer showed that the ibuttons consistently record temperatures <1°C higher than these other thermometers. Therefore ibutton temperatures recorded over the course of the experiment were adjusted for consistency with the temperature measurements from the other experiments, which were measured using the Omega Thermometer. The effect of temperature on righting time was analyzed using an ANOVA in the Fit Model menu in JMP 11 with temperature treatment as a 4‐level fixed factor. The distribution of the residuals was compared with a normal distribution to verify model adequacy, using a Shapiro‐Wilk W test. Righting times were log‐transformed to conform to the homogeneity of variance tested with a Levene's test. The nominal logistic model was used to test for the effects of the predictor variable, experimental temperature, on binary response variables (survival 12 h after heating trial and movement immediately after exposure). L_50_ was estimated using the inverse prediction option. *P*‐values less than 0.05 were considered significant.

### Tolerance of fertilization and early development to acute stress

Thermal tolerance of fertilization and early development was tested using a thermal gradient generated by a heatblock (Sewell and Young 1999; Kuo and Sanford 2009). The heatblock is a custom‐made aluminum block with 10 rows and 4 columns of evenly spaced holes that snuggly fit 15‐mL scintillation vials. The temperature gradient was controlled by pumping water from temperature‐controlled waterbaths through each end of the block. Urchins were spawned following standard methods (Strathmann 1987) and a solution contained 200 eggs on average was allocated to each 15‐mL scintillation vial. Each vial contained 10 mL of 0.45 *μ*m filtered seawater. Eggs from a single female were used in each row. Each vial containing eggs was allowed 45 min to gradually come to equilibrium with the temperature in the surrounding block. At this time, the temperature was recorded with Omega High Accuracy Digital Thermometer. Mixed sperm from three males was added to each vial of eggs at a concentration in excess of what should guarantee 100% fertilization (~ 10 × 10^4^ sperm mL^−1^) (Farley and Levitan 2001), and at which we did not observe indications of polyspermy. The collected sperm was not acclimated to the experimental temperatures to avoid reduction in viability, therefore this assay shows the temperature at which the eggs are fertilizable rather than investigating the relationship between sperm concentration, temperature acclimation and sperm viability. The vials were kept in the heatblock for 2 h after the sperm were added. The temperature was measured again and the contents of the vials were then fixed in 2% formalin. Two replicate subsamples of 30 eggs were scored for the presence/absence of a fertilization envelope, initiation of cleavage and the attainment of the morula (≥16 cells). Eggs from 13 females were assayed in this way. The effect of temperature and female on successful attainment of each developmental landmark was tested using the Nominal Logistic option in the Fit Model menu in JMP 11. The binary response was successful completion of the developmental stage (fertilization, early cleavage, and morula), the predictor variables were temperature (continuous), female (categorical), and the interaction between female and temperature. We included female and the interaction between female and temperature as maternal effects could alter thermal tolerance. L_50_, L_25_, and L_10_ were estimated using the inverse prediction option. *P*‐values less than 0.05 were considered significant. We report L_10_, L_25_, and L_50_, as even small reductions in survival could be biologically meaningful. It should be noted that the L_50_ if the standard metric for studies of stress responses (de Vries et al. 2008) and that predictions around the upper asymptotes may vary depending on the model selected and are therefore less comparable than the L_50_ values (Ritz 2010).

*Larval Tolerance of Acute Stress* ‐ To determine the tolerance of larvae to acute stress larvae were raised at 28–29°C in 0.45 *μ*m filtered seawater with salinity of 36 psu. Larvae kept at a density of 1 larva per mL and were fed a diet of *Isochrysis* strain *T‐iso* until they reached the mature 4‐armed pluteus stage (3–4 days). Larvae were either raised in mixed cultures derived from eggs of three females and sperm of three males, or larvae from individual females were reared separately and then mixed in equal proportions prior to the experiment. Ten or twenty larvae were counted into each scintillation vial with 10 mL filtered seawater and the vials were incubated in the heatblock for 2 h. The temperature was measured as the vial was removed and the larvae were counted again and scored as alive or dead immediately after removal from the block. Statistical analysis was performed using the same logistic regression model as described above, but with culture date as a predictor variable instead of mother, as each culture was derived from mixed parents.

### Larval tolerance of chronic stress

To understand thermal tolerance of *L. variegatus* larvae to long‐term exposure, we reared larvae for 6–10 days under a range of temperatures. In 2014, we conducted two experiments where larvae were raised over a wide range of temperatures from 23–32°C. In 2015, we repeated this twice more with temperatures from 28–36°C to further refine our estimates of the temperatures at which reduced performance (i.e., growth and survival) and increased mortality are observed. Temperature treatments were planned in increments of 2 or 3°C, but ibuttons placed in the heated aquaria used to maintain the experimental temperatures indicated that the target temperatures were not always realized. Average temperatures actually measured in the treatments are reported rather than the target temperatures.

In 2014, fertilized eggs were placed into the beakers at a density of approximately 1 individual mL^−1^ and placed in waterbaths at the target temperatures. Larvae were reared in 0.45 micron‐filtered water in unstirred 1‐L beakers with six replicate beakers per treatment. Larvae were kept with a 12‐h light/dark cycle, water was changed every other day, 30 mg L^−1^ streptomycin antibiotic was included and larvae were fed 50,000 cells/mL *Isochrysis* daily. Larvae from 2, 10 mL samples were pooled, counted, and photographed every other day and measured on Day 4 after fertilization with Image J. Because fertilization success was sometimes well below 100%, cultures initiated in 2015 were maintained at 10 individuals mL^−1^ at the treatment temperatures until they hatched. After 24 h, they were allocated into the rearing beakers at densities of 1 individual mL^−1^. Larvae were fed a mix of *Isochrysis* and *Tetraselmis* sp, but otherwise conditions were the same as those in 2014.

Each experiment was analyzed separately due to differences in the realized temperature treatments. A multivariate approach as implemented with the repeated measures MANOVA personality in the Fit Model menu in JMP12 was used to account for repeated measures nature of our data. If the chi‐square test for sphericity was not significant, we report the unadjusted *F* tests, when it was significant, we report the Greenhouse‐Geisser adjusted *F* tests.

### Environmental data

The results of our temperature tolerance assays were compared with two environmental datasets collected over the last 10 years in Bocas del Toro by STRI's Physical Monitoring Program. From 2005–2015, temperature was recorded 2 m off the bottom (approximately 2–3 m depth) on the instrument platform at the Bocas del Toro Research Station (BRS platform, hereafter), at 15‐min intervals by data‐loggers attached to a Campbell Model 107 electronic temperature sensor. The platform is located over the seagrass bed where adult *L. variegatus* were collected for this study. Temperature was also recorded hourly at 3 m depth on a shallow, protected reef near Isla Pastores (9°13′11″N, 82°19′31″W) using a HOBO Stow‐Away TidbiT and HOBO Water Temperature Pro V2 (Onset Computer Corporation, Bourne, MA) with an accuracy of ±0.25 °C (Kaufmann and Thompson 2005). This area is particularly protected and is somewhat warmer than more open sites around the Bocas del Toro Archipelago. Sensors were changed and calibrated biannually.

## Results

### Adult tolerance of acute stress

**Figure 1:**
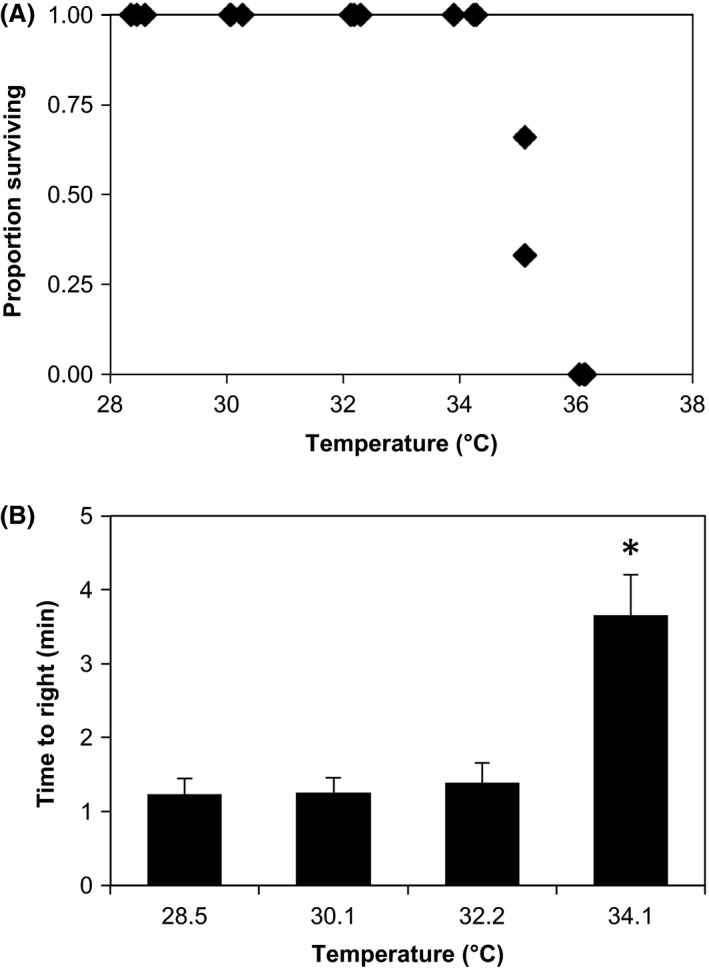
Response of adult *L. variegatus* to 2‐h temperature exposures. (A) Logistic regression showing the effect of 2‐h temperature exposures on survival 24 h postexposure (*L*_50_ = 35.1°C; c.i. = 34.8–35.4°C). Each point represents an independent trial with three adult urchins. Three trials were made at each temperature. Overlapping points are not visible. (B) Bar graph showing the results of ANOVA analysis of effects of temperature on righting time (ANOVA:* r* ^2 ^= 0.48; *n* = 36; df = 3; *F* = 9.93; *P* < 0.0001). Error bars indicating the standard error and * indicates significant difference at *P* < 0.0001.

All adult urchins assayed survived 2‐h exposures in our 28°C to 34°C treatments, but none survived the 36°C treatment. Three of the six animals exposed to 36°C showed some slight reflexive movement of the spines at the end of the 2‐h exposure but they failed to right themselves or attach to the substrate, and they all died within 24 h of the exposure. At 35°C, no animals could right themselves after the 2‐h exposure, but four of nine animals tested were alive 24 h later. Logistic regression shows that the LT_50_ for 24‐h survival after the 2‐h exposure was 35.1°C (34.8–35.3) (Fig. 1A; Tables 1, 2) and for movement immediately after exposure was 36.1°C (36.1–36.2), indicating that righting behavior slightly underestimates survival and small movements overestimates long‐term survival after 2‐h exposures. The negative impact of heating appeared rapidly and it is clear that the tipping point is between 34°C and 36°C. However, sublethal negative effects of heating were evident in the time to righting. ANOVA with post hoc Tukey HSD tests shows no significant difference between righting times of urchins exposed to 28°C, 30°C, and 32°C, but a significantly longer righting time at 34°C (Fig. 1B; *r* ^2 ^= 0.50; *N* = 36; df = 3; *F* = 10.75; *P* < 0.0001). There was no significant difference between righting times of the urchins assigned to each treatment before the exposure (*r* ^2 ^= 0.12; *N* = 36; df = 3; *F* = 1.43; *P* = 0.25).

**Table 1:** LT_50_, LT_25_, and LT_10_ and 95% confidence intervals of the inverse predictions for fertilization, initiation of cleavage, and development to morula, as well as larval survival and adult survival after exposure to 2 h of acute temperature stress

Stage	LT_10_	LT_25_	LT_50_
Fertilization	33.0°C (32.9–33.1)	33.7°C (33.6–33.8)	34.4°C (34.3–34.5)
Cleavage	32.6°C (32.5–32.7)	33.0°C (33.0–33.1)	33.5°C (33.4–33.5)
Morula	31.8°C (31.8–31.9)	32.2°C (32.1–32.2)	32.5°C (32.4–32.5)
Larvae	32.1°C (31.9–32.2)	33.1°C (32.9–33.2)	34.1°C (34.0–34.2)
Adults	34.9°C (NA)	35.0°C (33.6–36.4)	35.1°C (34.8–35.6)

**Table 2:** Nominal logistic regression of five life stages of *L. variegatus* to acute thermal stress. Analyses were performed for the probability of (1) successful fertilization; (2) cleavage; and (3) development to a morula from fertilizations in the heatblock; (4) survival of 3–4 day old larvae after 2‐h exposure to acute thermal stress in the heatblock; and (5) survival of adults 24 h after 2‐h exposure to acute thermal stress in a water bath

Factor	df	Chi‐squared	*P*‐value
*1. Fertilization*			
Temperature	1	6392.47	**<0.0001**
Female	12	265.85	**<0.0001**
Temperature x Female	12	59.37	**<0.0001**
Generalized *r* ^2^	0.85	Converged after 9 iterations	
*2. Cleavage*			
Temperature	1	6960.17	**<0.0001**
Female	12	93.08	**<0.0001**
Temperature × Female	12	108.58	**<0.0001**
Generalized *r* ^2^	0.91	Converged after 23 iterations	
*3. Morula*			
Temperature	1	24324.28	**<0.0001**
Female	12	77.79	**<0.0001**
Temperature × Female	12	58.92	**<0.0001**
Generalized *r* ^2^	0.93	Converged after 24 iterations	
*4. Larvae*			
Temperature	1	1215.24	**<0.0001**
Culture date	6	85.46	**<0.0001**
Temperature × date	6	39.94	**<0.0001**
Generalized *r* ^2^	0.69	Converged after 8 iterations	
*5. Adults*			
Temperature	1	49.44	**<0.0001**
Generalized *r* ^2^	0.88	Converged after 17 iterations	

Bold values highlight statistically significant values.

### Acute thermal tolerance of fertilization and early development

**Figure 2:**
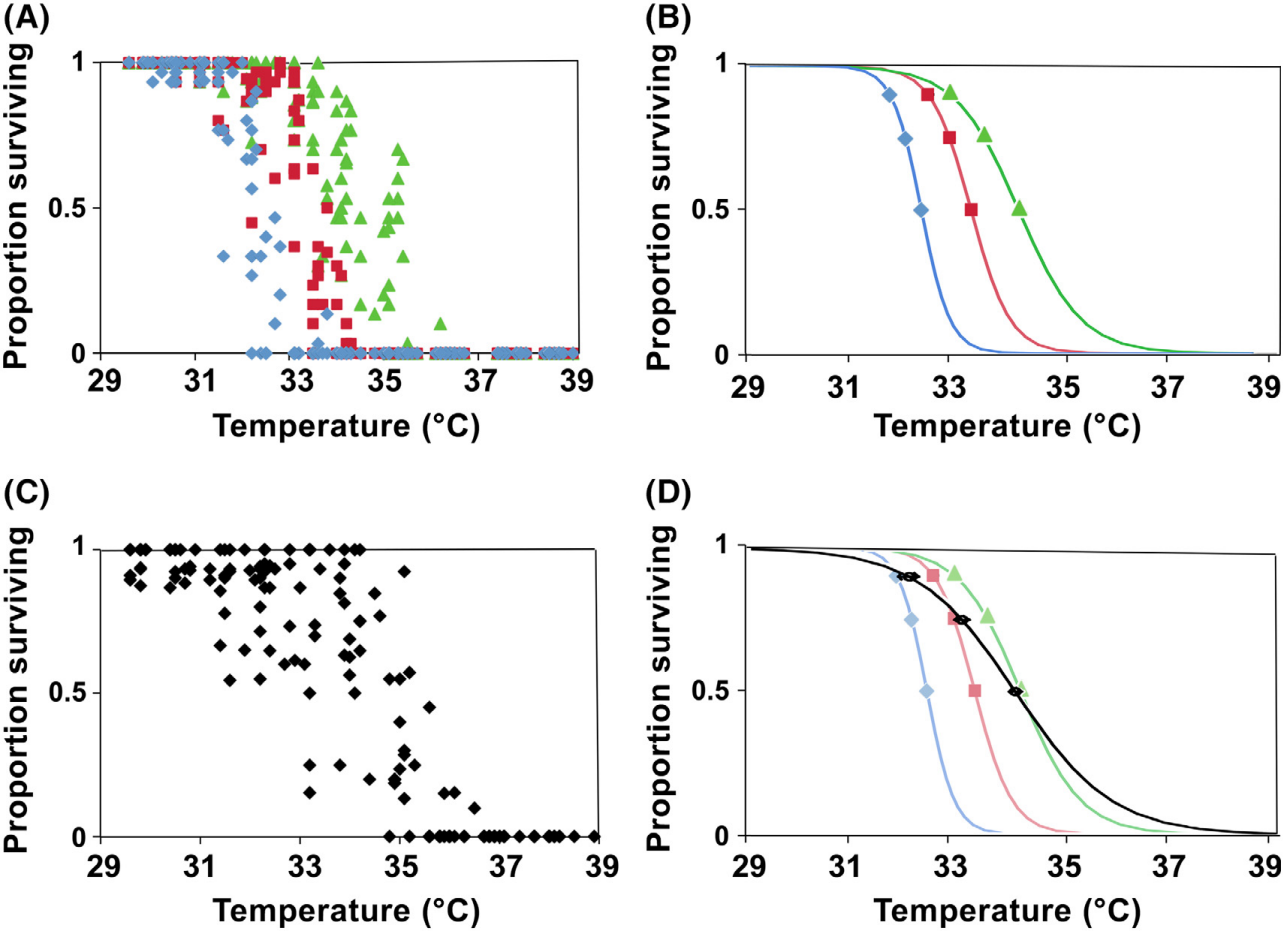
Data (A, C) and logistic regression (B, D) for temperature dependence of development from the heatblock assay of early development (A, B) and heatblock assay of 3–4 day old larvae (C, D). Each point represents the % survival of 60 embryos or 10–20 larvae counted from a single vial in one of nine replicate trials that placed 10 vials across the temperature gradient. Fertilization success (green triangles); initiation of cleavage (red squares); successful development to morula (blue diamonds), and larval survival (black ovals). Points on regression lines indicate 10%, 25%, and 50% mortality and horizontal lines extending from the points indicate the 95% confidence intervals (when visible).

For all females assayed, fertilization and normal development to morula was >90% at the coolest temperatures and no fertilization occurred at the highest temperatures (Fig. 2). Fertilization is more tolerant of high temperatures than is the initiation of development as for each female, moderately high temperature treatments which did not show cleavage did produce a fertilization envelope. Likewise a number of eggs initiated cleavage at temperatures at which development did not reach the morula stage. Logistic GLM analysis of our counts of fertilized, cleaving, and successfully developing embryos each showed a significant effect of temperature, female, and an interaction between temperature and female on the number succeeding to reach each of the three developmental stages (Table 2). The LT_50_ for fertilization ranged from 33.1°C to 35.2°C across the 13 different females. The LT_50_ for the initiation of cleavage ranged from 32.8°C to 33.9°C and for successful development to a morula or beyond 31.9°C to 32.9°C (Fig. 2A and B; Table 1). The LT_50_ for fertilization was not correlated with LT_50_ for cleavage (*P* > 0.1; *n* = 13) or later development (*P* > 0.1; *n* = 13) across females. The LT_50_ of cleavage was correlated with LT_50_ for successful development to morula (r = 0.54; *P* = 0.05; *n* = 13). However, this is primarily due to the low LT_50_‐values for one female. The overall LT_10_, LT_25_, and LT_50_ calculated across data from all the females combined show a progressive decrease during early development (Fig. 2; Table 1).

### Larval tolerance of acute stress

Assays of larval survival of a 2‐h exposure to acute thermal stress were more variable than the assays of early development, but nevertheless produced consistent LT_50_ values. Across the different rearing experiments, LT_50_ values ranged from 33.3–34.7°C. The overall LT_50_ of 34.1°C is significantly lower than the LT_50_ for adults and higher than the L_50_ for cleavage and morula development (Fig. 2C and D; Table 1). The L_25_ and L_10_ values are similar to those observed for cleavage and morula, respectively.

### Larval tolerance of chronic stress

Early larval development of *L. variegatus* proceeded normally over a range of temperatures from 23°C to 31.9°C. In experiments 3 and 4, we tested warmer temperatures and found there was 0% survival to day 1 at 32.3°C (SD = 0.1), 34.0°C (SD = 0.4), 35.8°C (SD = 0.7), and 35.9°C (SD = 0.6). This is consistent with the results from the heatblock assays that temperatures above 32°C significantly reduced survival to the morula stage.

**Figure 3:**
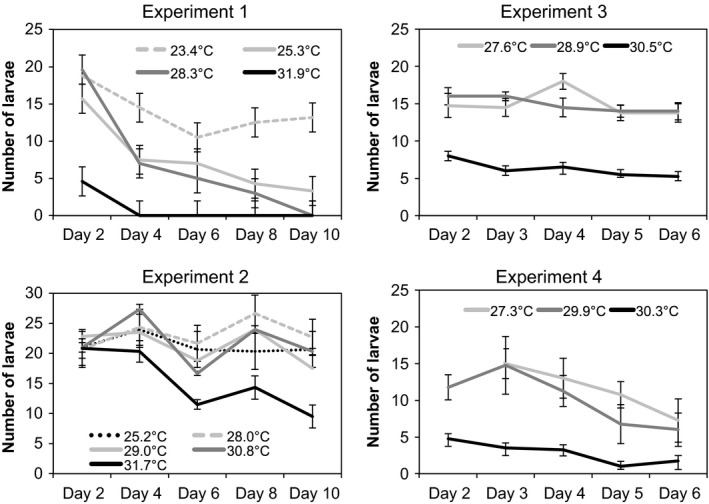
Relationship between culturing temperature and larval survival compared with days after fertilization. The number of larvae is the total number from two pooled 10‐mL samples. Temperatures (standard deviations) of the treatments are as follows. *Experiment 1*: 23.4°C (0.3), 25.3°C (0.1), 28.3°C (0.2), and 31.9°C (0.1). *Experiment 2*: 22.8°C (0.4), 25.2°C (0.2), 28.0°C (0.1), 29.0°C (0.2), 30.8°C (0.3), and 31.7°C (0.4). *Experiment 3:* 27.6°C (0.2), 28.9°C (0.2), and 30.5°C (0.4). *Experiment 4*: 27.3°C (0.2), 29.9°C (0.2), and 30.3°C* (0.1). *This treatment drifted over time with an average temperature of 30.8°C for the first 2 days and 30.0°C thereafter.

Counts of larval survival through time showed consistent results across the four experiments. In all cases, larval survival was highest at cooler temperatures (Fig. 3; Table 3). Differences in survival had already appeared by the first day we sampled the cultures (Day 2) except for in Experiment 2 in which all cultures showed similar survival to Day 2. In Experiment 1, the 31.9°C cultures survived to Day 2, but crashed suddenly between Day 2 and Day 4 (Fig. 3). MANOVA of each experiment showed a significant effect of temperature treatment in all cases (Table 3). Experiments 1, 2, and 4 showed a significant effect of day, reflecting a slow decrease in the number of larvae over time, but such a decrease was not evident in Experiment 3. Experiment 1 also showed a significant interaction between temperature and day, with larvae reared at 31°C showing a more rapid decrease and those reared at 23.4°C a slower decrease in survival over time compared with those raised at other temperatures (Table 3; Fig. 3). Pairwise post hoc tests showed that for Experiments 3 and 4, larvae in the warmest treatment had significantly lower survival than the other two treatments. In Experiment 2, the warmest treatment (31.7°C) had significantly lower survival than 29°C and 28°C but did not differ significantly from 30.8°C and 25.2°C. Overall, larvae reared above 30°C had lower survival than those reared at cooler temperatures. This effect often occurred during the first 2 days, and we recorded similar loss rates between the 30°C and other temperatures as evidenced by nonsignificant interactions between day and temperature after that. The exception to this generalization is that in Experiment 2 larvae reared at 30.8°C survived as well as those reared at cooler temperatures, and some of those reared at 31.9°C survived for several days. Experiments 1 and 2 took place when field temperatures where warmer than when we conducted Experiments 3 and 4, which may account for the better early survival of larvae reared at the higher temperatures in the first two experiments.

**Table 3:** Repeated measures analysis of larval survival in cultures at different temperatures on 5 days after fertilization. Bold highlights statistically significant *P*‐values

	*F*	NumDF	DenDF	*P*
*Experiment 1 (May)*				
Temperature	10.09	3	20	**0.0003**
Time	31.09	2.15	43.02	**<0.0001**
Time × Temperature	2.60	6.45	43.02	**0.03**
*Experiment 2 (June)*				
Temperature	4.91	4	16	**0.0089**
Time	5.28	4	13	**0.0095**
Time × Temperature	1.02	16	64	0.44
*Experiment 3 (March)*				
Temperature	12.83	2	9	**0.002**
Time	0.68	3	7	0.59
Time × Temperature	0.42	6	27	0.85
*Experiment 4 (March)*				
Temperature	8.86	1	9	**0.008**
Time	7.65	1.73	15.53	**0.006**
Time × Temperature	1.01	3.45	15.53	0.42

**Figure 4:**
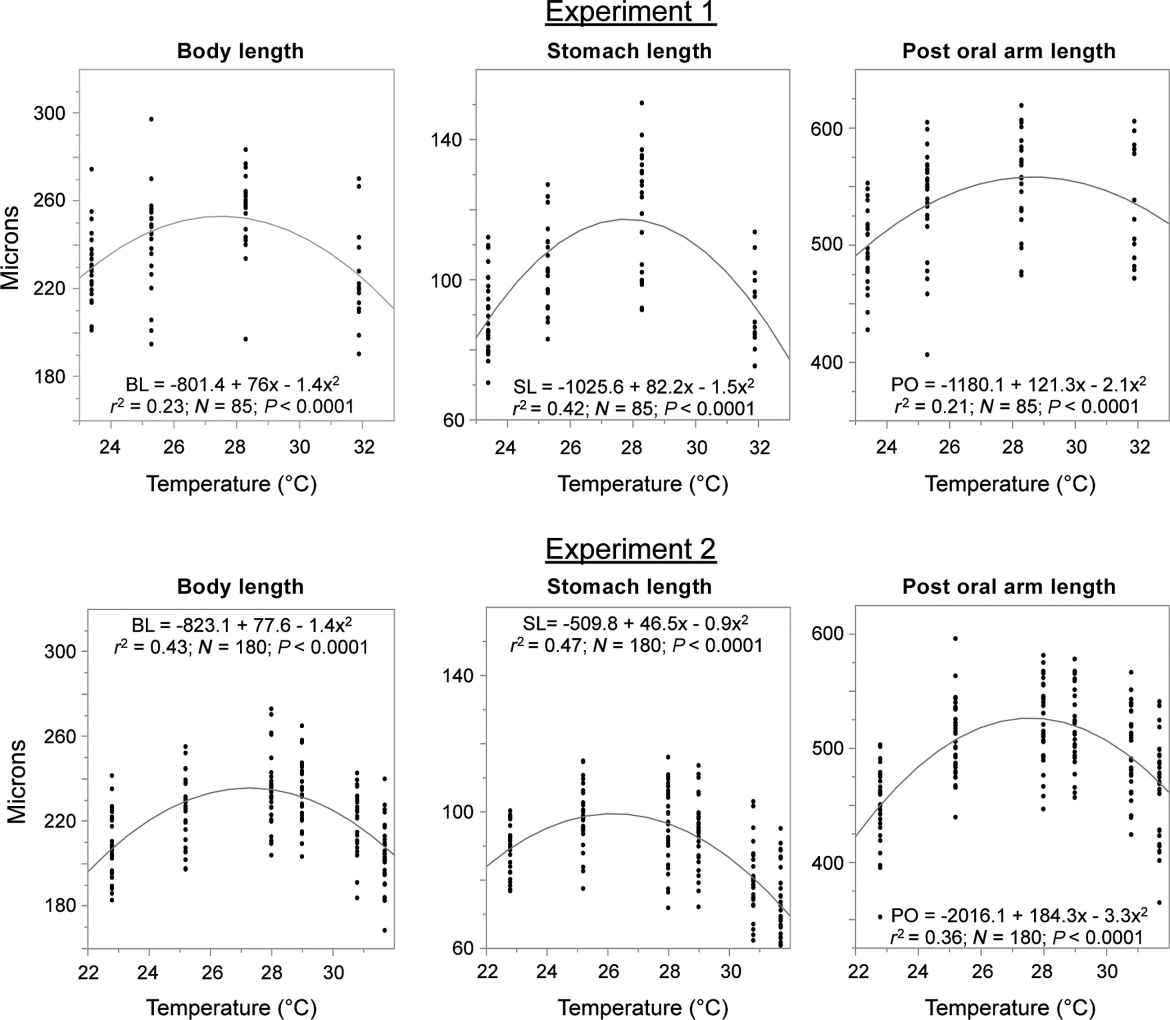
Relationship between culturing temperature and total length (from posterior end to the top of the oral hood), stomach length and total length of postoral arms (from posterior end to tip of postoral arm, averaged for both sides). Larvae from Experiments 1 and 2 were photographed on Day 4 after fertilization. Dots represent individual measurements and lines show the best fit quadratic, which strongly indicates a reduction in size above 29°C. In all cases, ANOVA analysis showed a significant effect of temperature on size and the results of the post hoc Tukey HSD tests indicated that larvae reared at 31.9°C in Experiment 1 and at 30.8°C and 31.7°C in Experiment 2 had significantly smaller body lengths and stomach length than larvae raised at 26–29°C.

Rearing temperature also had a significant impact on larval size (Table 4). Measurements of larval body length, stomach length, and postoral arm length on 4‐armed plutei all varied in concert (Fig. 4). From 23–30°C, each of these measurements either increased with temperature or remained the same. Above 30.5°C, all three lengths were reduced compared with the cooler temperature treatments (Fig. 4).

**Table 4:** One‐way ANOVA analysis testing for effect of rearing temperature on three measures of larval size. Larvae from Experiments 1 and 2 were measured on Day 4 after fertilization and those from Experiments 3 and 4 were measured on Day 3 after fertilization (see methods for details). Bold highlights statistically significant *P*‐values

Factor	df	*F*	*P*‐value
*Experiment 1 (May)*			
Total length	3	22.4	**<0.0001**
Stomach length	3	22.6	**<0.0001**
Postoral arm length	3	7.48	**0.0002**
*Experiment 2 (June)*			
Total length	5	25.8	**<0.0001**
Stomach length	5	31.0	**<0.0001**
Postoral arm length	5	20.4	**<0.0001**
*Experiment 3 (March)*			
Total length	2	73.7	**<0.0001**
Stomach length	2	17.5	**<0.0001**
Postoral arm length	2	31.2	**<0.0001**
*Experiment 4 (March)*			
Total length	2	9.4	**0.0005**
Stomach length	2	4.9	**0.01**
Postoral arm length	2	10.7	**0.0002**

### Environmental data

Biologically meaningful reduction in successful development occurs at temperatures experienced each year in Bocas del Toro. Monthly average water temperatures recorded at the BRS instrument platform ranged from 27.5°C in January to 29.7°C in September and near Isla Pastores ranged from 28.2°C in January to 30.6°C in October (Table 5). These are similar to the temperatures reported for other sites around Bocas del Toro (Kaufmann and Thompson 2005; Collin et al. 2009; Neal et al. 2014). Between 2005 and 2015, the BRS platform showed that temperatures exceed 30°C frequently during May, June, August, and September. In some years, temperatures exceed 31°C in those months but were never reported to exceed 32°C. The Pastores site was warmer and temperatures exceeded 31°C for 456 h on average each year. In addition, in 2003, 2005, and 2010 temperatures exceeded 32°C on several occasions.

**Table 5:** Average monthly temperatures at 2 shallow‐water sites in Bocsa del Toro. Measurements were taken every 15 min from the BRS platform from 2006–2015, and hourly near Isla Pastores from 1999–2014. The average number of time periods with temperatures above 30°C, 31°C, and 32°C per month

Month	BRS Platform				Isla Pastores			
	Average Temperature (°C)	Average number of hours >30°C	Average number of hours >31°C	Average number of 15‐min periods >32°C	Average Temperature (°C)	Average number of hours>30°C	Average number of hours >31°C	Average number of hours>32°C^a^
January	27.6	0	0	0	28.2	0.6	0	0
February	27.7	0	0	0	28.3	0	0	0
March	28.1	0.15	0	0	28.6	32.1	0	0
April	28.8	27.8	0	0	29.3	68.1	0	0
May	29.5	122.8	1.4	0	29.8	344	12.7	0
June	29.6	147.2	4	0	30.2	463.1	95.6	3.6
July	29.0	27.4	0	0	29.6	180.9	18.1	0
August	29.3	68.4	0.6	0	29.7	310.8	42.3	0
September	29.7	195.2	4.2	0	30.4	533.7	153.9	20.1
October	29.7	213.8	0.23	0	30.6	537.2	128.3	1.1
November	28.5	16	0	0	29.2	143.7	7.2	0
December	27.7	0	0	0	28.2	8.1	0	0

a Temperatures only exceeded 32°C in 2003, 2005 and 2010.

## Discussion

Our assays of tolerance of acute thermal stress using a standardized 2‐h exposure across five stages of the urchin development show that thermal tolerance of acute stress does vary among life stages. Both these acute assays and tests of chronic thermal stress on larval growth and survival suggest that *L. variegatus* may already be exposed to temperatures near the thermal limit for successful development, larval survival, and recruitment, indicating significant risk under current global warming scenarios.

Our work is one of a few to systematically determine what stage of the sea urchin life cycle is the most vulnerable to thermal stress, testing the general belief that early developmental stages are more susceptible to stressors than are later stages (Byrne 2012; but see Hamdoun and Epel 2007; Byrne et al. 2010; Tangwancharoen and Burton 2014). Results from our experiments indicate that cleavage and early embryonic development are more susceptible to acute thermal stress than are fertilization or adults. The 4‐armed larval stage is also less susceptible to thermal stress than cleavage or early embryonic development when assessed using L_50_ as a measure of susceptibility. Other work with tropical and subtropical sea urchins using similar assays to examine fertilization success and early development show similar results, suggesting sensitivity of certain life stages may be consistent across species. For example, in the Caribbean sea urchin *Echinometra lucunter*, a species broadly sympatric with *L. variegatus*, fertilization succeeds at temperatures up to 37°C and 4‐armed larvae can survive up to 39°C, but early embryonic development is successful only up to 34°C (Sewell and Young 1999). In the subtropical Australian sea urchin *Heliocidaris erythrogramma,* fertilization also occurs at temperatures too high for embryonic development. Adults collected at 20°C produced eggs with fertilization success of 90% or greater at 24°C and 26°C, but only 50% of the embryos develop to gastrulae at 24°C and fewer than 10% develop to gastrulae at 26°C (Byrne et al. 2009). In case of *L. variegatus,* adults have higher tolerances to 2‐h exposures to elevated temperatures than do any of the developmental stages that we examined. Comparable data on adults are not available for these other species.

Our larval growth experiments suggest that low larval survival at chronic exposures to 30–32°C is due to reduced early survival, and that once larvae reach the 4‐armed stage (~2 days in this experiment) mortality rates are similar to larvae raised at lower temperatures. This is also consistent with previous work on *Echinometra lucunter* which showed that embryos that completed early development at cool temperatures could survive well when later transferred to temperatures too high for early development (Sewell and Young 1999). This suggests that at 30–32°C those *L. variegatus* embryos that do survive the early critical period can survive subsequent development. However, we found that larvae raised at high temperatures were significantly smaller than those raised at lower temperatures. Whether this is due to the larval rearing temperature or to carry‐over effects from stress experienced at the early developmental stages is not clear. Understanding the impact of short‐term exposures to high temperatures and their impacts on later stages is an important area for future investigation.

Our data suggest that *L. variegatus* currently live close to the thermal limit for successful reproduction. Environmental monitoring at the Isla Pastores site, which is an enclosed bay deep within Bahia Almirante, but which supports reef and seagrass ecosystems, shows that average water temperatures exceed 30°C for 2 months of the year. In warm years, temperatures can exceed 31°C for several days and can even sometimes exceed 32°C. Our results show that short‐term exposures to such high temperatures do not impact adult *L. variegatus* survival or righting responses. However, work with the same species collected from the Gulf of Mexico shows that 10‐h exposures as well as chronic exposures to 32°C have a strong negative effect on righting and covering performance (Brothers and McClintock 2015). In addition, embryonic survival and larval growth are significantly impacted above 30.5°C. As *L. variegatus* reproduces all year round along the Caribbean coast of Panama (Lessios 1984; pers. obs.), it is likely that reproductive success of urchins at some sites in the Bocas del Toro archipelago is already reduced during these warm times of the year.

The exposure risk of thermal stress to early developmental stages depends significantly on the rate at which they are exported from the warm, shallow seagrass beds to cooler, more open water. While we do not know how spawning is timed with respect to daily temperature fluctuations and with the tidal cycle, modeled flow velocities in the Bahia Almirante are slow, with retention times of >20 h (Li and Reidenbach 2014). This suggests that newly spawned embryos could likely be retained in warm shallow water for over 2 h and experience significant thermal stress. A circulation model of sea surface temperatures for the region predicts that by 2054 most of the Bahia Almirante will experience temperatures of 31°C in August and that temperatures will be near 32°C for the same time period in 2084 (Li and Reidenbach 2014). Unless *L. variegatus* adapt or acclimate to these elevated temperatures, it is likely that these urchins will experience significantly reduced larval survival and possibly complete reproductive failure in much of Bocas del Toro by the end of this century. Although not designed to explicitly address this topic, our experiments suggest possibility of genetic variation in thermal tolerance. The eggs from individual females showed significantly different L_50_ for fertilization, cleavage and successful development to morula, as well as highly significant female X temperature effects. These results could reflect genetic differences in thermal tolerance, general stress responses, or developmental robustness among mothers, which could play an important role in adapting to future environmental change.

## Conflict of Interest

None declared.
